# Editorial: Prevention and early detection of oral cancers – volume II

**DOI:** 10.3389/froh.2026.1972756

**Published:** 2026-09-22

**Authors:** Abhimanyu Kumar Jha

**Affiliations:** Department of Biotechnology and Bioengineering, School of Biosciences and Technology, Galgotias University, Greater Noida, India

**Keywords:** early detection, HPV-associated oral lesion, optical imaging, oral squamous cell carcinoma, tobacco risk factor

Oral Squamous Cell Carcinoma (OSCC) remains the most common type of oral cancer, primarily originating from the epithelial lining of the oral cavity, and continues to represent a significant global health concern with persistently high mortality despite the accessibility of the oral cavity to clinical examination (1). The occurrence of diagnostic delay, caused by clinical overlaps with non-cancerous diseases and drug-related adverse events, plays a crucial part in determining prognosis and outcomes of the disease (2). In this Research Topic entitled Prevention and Early Detection of Oral Cancers**–** Volume II, five different contributions will cover different topics within the area, such as diagnostic overlap in OSCC and MRONJ in patients treated with bone-modifying agents, HPV-associated lesions, imaging technology in oral oncology, epidemiology of risk factors, and educational efforts in early oral cancer detection. Five different articles will discuss different, yet related, aspects of the topic, namely diagnostic overlap in OSCC and MRONJ in patients receiving bone-modifying drugs, diagnosis and management of HPV-related oral lesions, optical imaging in oral oncology and OPMDs, epidemiology of smoking as a risk factor for developing oral and pharyngeal cancer, and an educational intervention in the diagnosis of oral cancers.

A central diagnostic challenge addressed in this volume concerns the differentiation of OSCC from conditions that closely mimic its presentation. According to Mauceri et al., a group of eleven patients treated with bone modifying agent or anti-angiogenic therapies had OSCC showing similar clinical and radiological appearances as MRONJ. These eleven out of a total of 711 patients originally suspected of suffering from MRONJ were eventually found to have OSCC based on results obtained through biopsy, with the mandible being the most common area of involvement. Some of the overlapping characteristics observed in the cases include bone exposure, mucosal defect, soft tissue swelling, and cortical erosion. This suggests that while biopsy may not be necessary for the diagnosis of MRONJ, biopsy should be considered for patients taking bone-modifying agents, even at a lower dose for osteoporosis treatment, when there is suspicion of malignancy (Mauceri et al.).

Another parallel problem in diagnosis is discussed by Anaya-Saavedra et al., presenting their brief discussion about HPV-OLs. In this review, the four types of HPV-OL, such as squamous papilloma, verruca vulgaris, condyloma acuminatum, and multifocal epithelial hyperplasia, are presented along with their distinct but commonly overlapping characteristics, making their classification difficult to identify. Two common myths about HPV-OLs are debunked in this paper. The first myth is about sexual transmission because of compelling proof that HPV-OLs can be transmitted horizontally, by autoinoculation, perinatally, or via fomites. Meanwhile, the second myth is about their malignant potential because these lesions are generally caused by low-risk HPV genotypes that are in the form of an episome and not chromosomal instability like that seen in dysplastic transformation. The paper concludes that conservative surgery remains the only treatment available, while topical medications used for dermatologic and anogenital treatments may cause harmful effects on the oral mucosa. Genotyping, too, cannot be considered as an effective diagnostic approach to detecting HPV-OLs. People with HIV are encouraged to undergo periodic surveillance because of high recurrences and genetic diversity of lesions in this subgroup (Anaya-Saavedra et al.).

Regarding future diagnostic techniques, Acharya et al. examine critically the potential use of various types of optical imaging (OI), including confocal reflectance microscopy, optical coherence tomography, narrow-band imaging, and Raman spectroscopy, in oral cancer and oral premalignant disorders (OPMDs) in general, specifically emphasizing OSF. The review is based on the presence of clearly identified ultrastructural and histologic alterations in OSF, such as progressive collagen deposition and remodeling, flattening of the epithelial-connective tissue junction, epithelial atrophy and dysplasia, and stage-specific changes in vasculature. While all these techniques have some level of validation for epithelial-based conditions like leukoplakia and OSCC, there are no data for OSF, with depth of penetration being the key limitation due to the presence of submucosal fibrosis in OSF progression stages. OI is considered to be more of an exploratory field for OSF, with extracellular matrix-oriented optical biomarkers and artificial intelligence (AI)-aided image analysis emerging as prospective directions needing correlation with pathology (Acharya et al.). This complex diagnosis is heavily based on the influence of modifiable risk factors. In this regard, Possenti et al. provide a systematic review and meta-analysis that examines the link between smoking and the risk of OPC. Based on an analysis of 115 original studies that included over 40,000 cases, current smokers had almost four times higher chances of developing OPC when compared to non-smokers (RR 3.58), whereas former smokers had a 60% increase in OPC risk (RR 1.61). According to dose-response analyses, OP risk doubles when smoking is only six cigarettes per day for over seven years of smoking, after which no further increase in risk is seen at higher smoking rates, whereas after quitting smoking, risk reduces consistently over time reaching half its level in about ten years. The effect of smoking on OPC risk was notably weaker among people who tested positive for HPV infection when compared with those who tested negative for HPV, and considerable geographical differences existed as well, with the lowest risks being associated with Asian populations (Possenti et al.).

Finally, translating risk-factor knowledge into clinical vigilance depends on the competency of front-line practitioners, a gap addressed by Chauhan et al. in a before-and-after quasi-experimental study evaluating an educational intervention for general dental practitioners across multiple regions of Indonesia. The intervention aimed to strengthen participants' ability to associate recognized oral cancer risk factors with corresponding pathological changes in the oral mucosa. Among 177 pre-intervention and 144 post-intervention responses, the correlation between correctly identified risk factors and pathological changes improved modestly from 0.464 to 0.485, though this difference did not reach statistical significance (*p* = 0.8109). The findings suggest that single educational sessions may be insufficient to produce measurable improvements in diagnostic reasoning, and that more comprehensive, repeated, or case-based training strategies may be necessary to translate theoretical knowledge into applied clinical pattern recognition (Chauhan et al.).

Collectively, the papers in this Research Topic illustrate that the problem of late diagnosis in oral cancer cannot be attributed to one barrier alone but rather multiple interrelated factors such as clinical overlap between oral malignancy and diseases like MRONJ, misunderstandings in relation to the biology of HPV-induced benign proliferations, a lack of maturity of non-invasive optical imaging techniques for fibrosis associated with pre-malignant processes like OSF, an established yet neglected risk relationship between dose and HPV effects for tobacco consumption, and insufficient diagnostic skills of general dentistry healthcare providers. The limitations of the present investigations include their small sample sizes due to case reports or *ex vivo* approaches, single-site and educational intervention trials. Meeting the larger challenges ahead involves vigilance and a low threshold for biopsy in suspicious cases, further development of imaging modalities, especially those based on optical and molecular techniques, and their validation in pathology-corrected disease-specific studies, continued efforts to combat tobacco consumption and regionally adjusted risk management, and education strategies going beyond simple classroom-based courses.

## References

[1] Muralidharan S Nikalje M Subramaniam T Koshy JA Koshy AV Bangera D. A narrative review on oral squamous cell carcinoma. J Pharma Bioallied Sci. (2025) 17(Suppl 1):S204–6. 10.4103/jpbs.jpbs_593_25 PMC12156633 40511239

[2] Ravindran S Ranganathan S R K J N A S Kannan SK. The role of molecular biomarkers in the diagnosis, prognosis, and treatment stratification of oral squamous cell carcinoma: a comprehensive review. J Liq Biop. (2025) 7:100285. 10.1016/j.jlb.2025.100285 PMC11863969 40027232

